# 15LO1 dictates glutathione redox changes in asthmatic airway epithelium to worsen type 2 inflammation

**DOI:** 10.1172/JCI151685

**Published:** 2022-01-04

**Authors:** Tadao Nagasaki, Alexander J. Schuyler, Jinming Zhao, Svetlana N. Samovich, Kazuhiro Yamada, Yanhan Deng, Scott P. Ginebaugh, Stephanie A. Christenson, Prescott G. Woodruff, John V. Fahy, John B. Trudeau, Detcho Stoyanovsky, Anuradha Ray, Yulia Y. Tyurina, Valerian E. Kagan, Sally E. Wenzel

**Affiliations:** 1 Department of Respiratory Medicine, Graduate School of Medicine, Kyoto University, Kyoto, Japan.; 2 Department of Environmental and Occupational Health, Graduate School of Public Health, University of Pittsburgh, Pittsburgh, Pennsylvania, USA.; 3 University of Pittsburgh Asthma and Environmental Lung Health Institute, Pittsburgh, Pennsylvania, USA.; 4 Department of Medical Oncology, Dana Farber Cancer Institute, Boston, Massachusetts, USA.; 5 Department of Medicine and Immunology, University of Pittsburgh School of Medicine, Pittsburgh, Pennsylvania, USA.; 6 Division of Pulmonary and Critical Care Medicine, Department of Medicine, UCSF, San Francisco, California, USA.

**Keywords:** Metabolism, Pulmonology, Asthma, Radicals, Th2 response

## Abstract

Altered redox biology challenges all cells, with compensatory responses often determining a cell’s fate. When 15 lipoxygenase 1 (15LO1), a lipid-peroxidizing enzyme abundant in asthmatic human airway epithelial cells (HAECs), binds phosphatidylethanolamine-binding protein 1 (PEBP1), hydroperoxy-phospholipids, which drive ferroptotic cell death, are generated. Peroxidases, including glutathione peroxidase 4 (GPX4), metabolize hydroperoxy-phospholipids to hydroxy derivatives to prevent ferroptotic death, but consume reduced glutathione (GSH). The cystine transporter SLC7A11 critically restores/maintains intracellular GSH. We hypothesized that high 15LO1, PEBP1, and GPX4 activity drives abnormal asthmatic redox biology, evidenced by lower bronchoalveolar lavage (BAL) fluid and intraepithelial cell GSH:oxidized GSH (GSSG) ratios, to enhance type 2 (T2) inflammatory responses. GSH, GSSG (enzymatic assays), 15LO1, GPX4, SLC7A11, and T2 biomarkers (Western blot and RNA-Seq) were measured in asthmatic and healthy control (HC) cells and fluids, with siRNA knockdown as appropriate. GSSG was higher and GSH:GSSG lower in asthmatic compared with HC BAL fluid, while intracellular GSH was lower in asthma. In vitro, a T2 cytokine (IL-13) induced 15LO1 generation of hydroperoxy-phospholipids, which lowered intracellular GSH and increased extracellular GSSG. Lowering GSH further by inhibiting SLC7A11 enhanced T2 inflammatory protein expression and ferroptosis. Ex vivo, redox imbalances corresponded to 15LO1 and SLC7A11 expression, T2 biomarkers, and worsened clinical outcomes. Thus, 15LO1 pathway–induced redox biology perturbations worsen T2 inflammation and asthma control, supporting 15LO1 as a therapeutic target.

## Graphical abstract

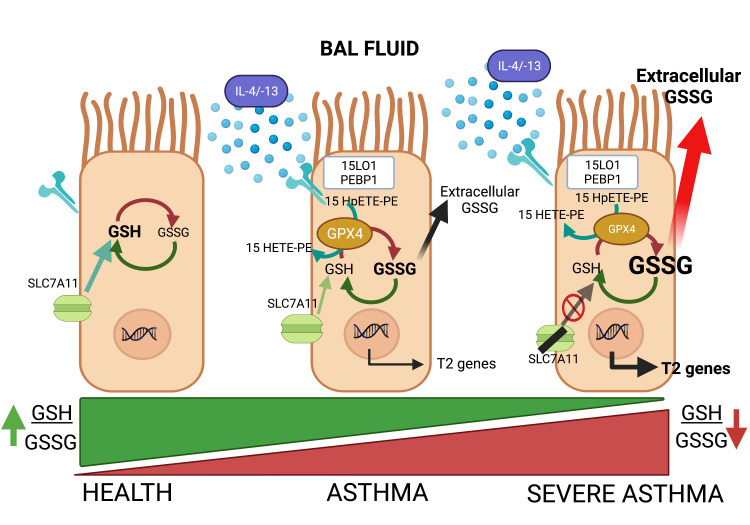

## Introduction

Among all internal organs, the lung is most consistently exposed to exogenous oxidant threats. Coupled with endogenous reactive oxygen species (ROS) generated as by-products of various cellular processes, strict regulation of antioxidants is critical to maintaining a balanced reduction/oxidation (redox) state. Reduced glutathione (GSH) is a ubiquitous antioxidant, utilized as a source of reducing equivalents by different GSH peroxidases, which requires constant intracellular GSH synthesis from cysteine, glutamate, and glycine (1, 2). GSH plays a pivotal role in maintaining intracellular redox balance by detoxifying peroxides and other oxidized compounds. Lower GSH levels and GSH to oxidized GSH ratio (GSH:GSSG), as measures of redox balance, have previously been reported in asthmatic lungs (3), although the external or internal immunoinflammatory pathways driving this dysregulation are unknown. Further, although several previous studies have evaluated GSH levels in various extracellular compartments, including bronchoalveolar lavage (BAL) fluid, these studies generally focused on alterations in GSH:GSSG balance in association with disease, or the consumption of GSH during prostaglandin or leukotriene synthesis, with more modest phenotypic, pathophysiologic, or mechanistic information (4, 5).

Although exogenous factors, including infection and inhaled pollutants, could directly oxidize GSH, endogenous disease-specific mechanisms could also prime the process. Under type 2 (T2) conditions (IL-13 stimulation), human airway epithelial cells (HAECs) generate oxidized phospholipids under control of the 15 lipoxygenase 1 (15LO1) enzyme and its binding with phosphatidylethanolamine-binding protein 1 (PEBP1) (6). These continuously generated electrophilic hydroperoxy-phospholipids are neutralized by a specific GSH-dependent peroxidase, glutathione peroxidase 4 (GPX4), which prevents them from damaging membranes and driving ferroptotic cell death (7, 8). In the absence of adequate GPX4, the radical intermediates, readily produced from hydroperoxy-phospholipids, directly oxidize GSH in a 1-electron reaction to generate a strongly pro-oxidant radical, GSH•. To limit potentially life-threatening depletion of intracellular antioxidant/GSH stores, epithelial cells have developed numerous compensatory pathways, including the cystine-glutamate antiporter (SLC7A11), which critically maintains de novo GSH biosynthesis by increasing intracellular cystine levels (9). Thus, under asthmatic conditions, intracellular GSH levels could reflect not only the generation of reactive hydroperoxy-phospholipids by the 15LO1-PEBP1 complex, but also the compensatory levels and activity of GPX4, the availability of newly synthesized and regenerated GSH, as well as extracellular transport. While failure of these compensatory mechanisms could ultimately cause ferroptotic cell death, more subtle changes in intracellular redox balance could also enhance signaling pathways, including those associated with T2 inflammation (10).

We hypothesized that the observed lower GSH:GSSG ratio in asthmatic lung fluids and cells is at least partially driven by high oxidation of GSH and its export in response to greater intracellular 15LO1/PEBP1 pathway activity. These lower intracellular GSH levels worsen T2 inflammation and asthma severity. To address this hypothesis, we measured BAL fluid and fresh intraepithelial cell GSH and GSSG levels in well-characterized asthmatic and healthy control (HC) participants. To mechanistically determine whether 15LO1 pathway activation alters the intracellular redox balance, particularly under T2 conditions, these ex vivo findings were modeled in vitro in primary HAECs. Finally, these in vitro findings were recapitulated ex vivo in 2 distinct asthma populations and their clinical relationships to asthma severity and control determined.

## Results

### Characteristics of the study population

BAL fluid samples were obtained from 23 HC, 32 mild-moderate asthma (M/M), and 43 severe asthma (SA) subjects (Table 1) recruited through either Severe Asthma Research Program 3 (SARP3) (HL-109152) or Immune Mechanisms of Severe Asthma (IMSA) (AI-06684). While the patient groups did not differ by sex, M/M were of slightly lower age relative to both HC and SA and, unsurprisingly, higher fractional exhaled nitric oxide (FeNO) and lower FEV_1_ (% predicted) were observed across groups from HC to SA. Bronchoscopically obtained fresh HAECs were analyzed from 11 HC, 4 M/M, and 11 SA subjects, a subset of the local IMSA cohort with matching BAL samples (Supplemental Table 1; supplemental material available online with this article; https://doi.org/10.1172/JCI151685DS1). The epithelial brushing subgroup did not differ from the full IMSA cohort. In vitro studies were performed on HAECs cultured at the air-liquid interface (ALI). Due to limited cell numbers available, the samples from the in vitro and ex vivo studies did not always overlap.

### The extracellular and intracellular redox balance, as measured by GSH and GSSG levels, differs between asthma and controls

#### BAL fluid.

**Figure 1:**
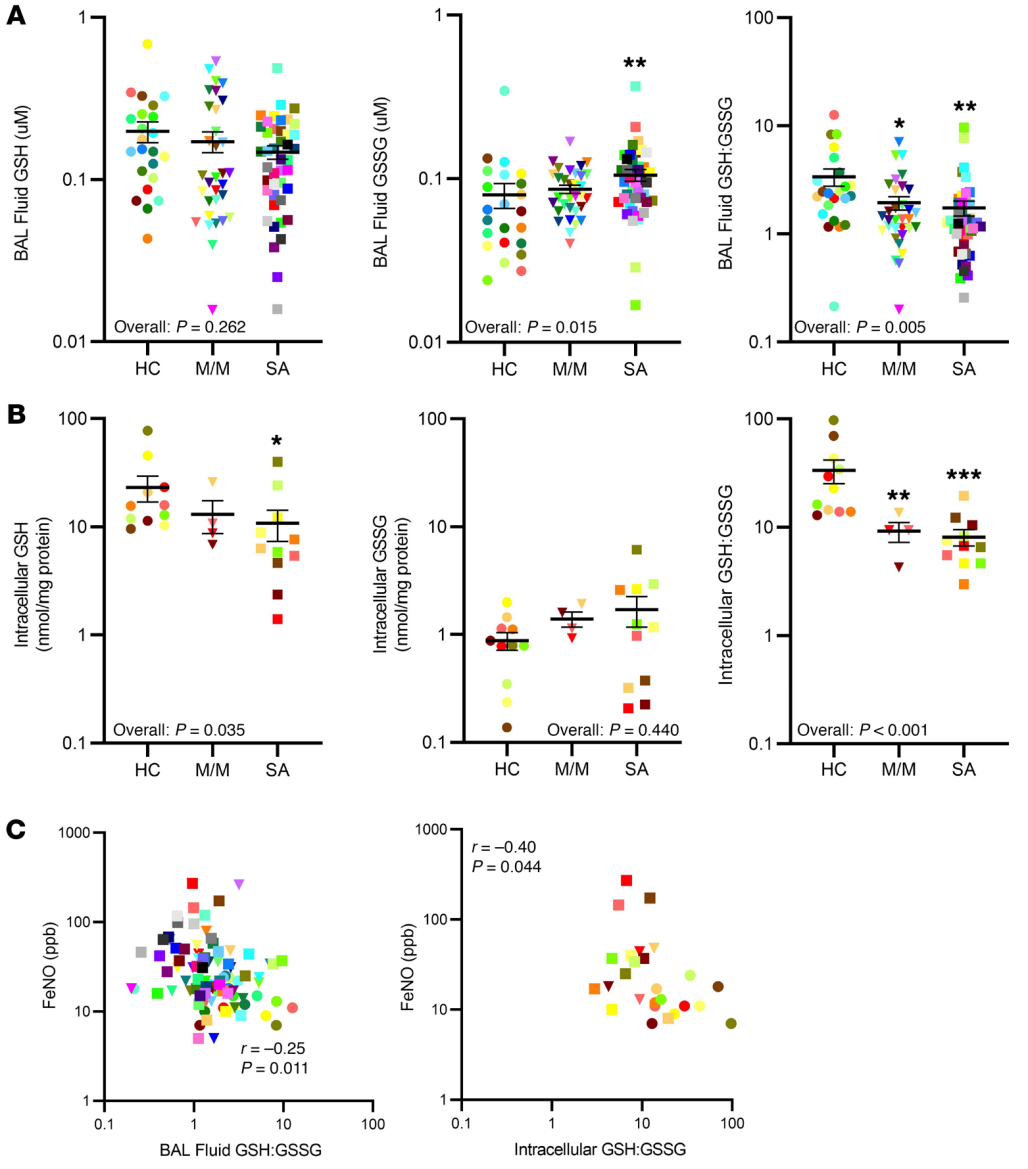
Abnormal redox balance, as measured by higher BAL fluid oxidized glutathione (GSSG) and lower intraepithelial cell GSH, is observed in asthmatic BAL fluid, as compared with healthy controls. GSH, GSSG, and the GSH:GSSG ratio were measured by enzymatic assay in bronchoalveolar lavage (BAL) fluid and in fresh epithelial cells from healthy controls (HCs), mild/moderate (M/M), and severe asthma (SA) patients. (**A**) Higher levels of GSSG and lower GSH:GSSG ratios were observed in asthmatic BAL fluid. (**B**) Intracellular GSH levels and GSH:GSSG were lower in fresh asthmatic epithelial cells compared with HCs. (**C**) BAL fluid (*n =* 98) and intracellular GSH:GSSG (*n =* 26) positively correlated with FeNO. ANOVA with intergroup comparisons by *t* test was used for group comparisons. Pearson’s correlations were used for GSH:GSSG versus FeNO. Bonferroni-corrected significant *P* values for 3 groups was set at 0.0166. All data were log transformed prior to analysis and were converted back to the linear scale for presentation. Bars represent mean and error bars represent SEM. *P < 0.01, **P < 0.001, ***P < 0.0001.

BAL fluid is an extracellular fluid compartment contributed to by both environmental and cellular factors. GSSG levels were higher in BAL fluid from SA as compared with that from HCs and were marginally elevated in M/M (overall *P =* 0.015). SA patients had higher BAL fluid GSSG than HCs (*P =* 0.004). These higher GSSG levels associated with lower GSH:GSSG ratios in BAL fluid (overall *P =* 0.004) (Figure 1A). BAL fluid GSH:GSSG was lower in both M/M (*P =* 0.020) and SA (*P =* 0.001) compared with HC. Although median levels were numerically lower in SA compared with M/M, there were no differences in BAL fluid GSSG or GSH:GSSG between M/M and SA. Controlling for BMI (significantly different across the participant groups) did not alter the results. GSH levels in BAL fluid did not differ across the 3 groups (overall *P =* 0.262). These data are in line with previously published data showing altered redox balance in asthmatic compared with healthy BAL fluid (3). FeNO is a well-recognized marker of T2 inflammation, being elevated in asthma and dramatically lowered by targeted T2/IL-4 receptor α antibodies (11, 12).

#### Intracellular.

To determine whether these BAL fluid levels were a reflection of intracellular GSH metabolism, freshly brushed and isolated HAECs were analyzed and compared to BAL fluid levels (Figure 1B). In contrast to BAL fluid, intracellular GSH (overall *P =* 0.035) was lower in SA (*P =* 0.010) compared with HC patients. Intracellular GSSG levels were low and did not differ between the groups (overall *P =* 0.440). However, similar to the BAL fluid, intracellular GSH:GSSG was substantially lower (overall *P <* 0.001) in both M/M and SA compared with HC (*P =* 0.004 and *P <* 0.001, respectively). Neither intracellular GSH nor GSH:GSSG differed between M/M and SA.

#### Relationship between the extracellular and intracellular compartments.

To determine whether BAL fluid redox measures reflected intracellular (HAEC) levels, overlapping samples were compared (*n =* 26). Intracellular GSH levels correlated with BAL fluid GSH levels (*r* = 0.41, *P =* 0.037). There were no correlations between intracellular and BAL fluid GSSG or GSH:GSSG (Supplemental Table 2). Of note, enzymatic GSH, GSSG, and GSH:GSSG measurements corresponded well with measures using a high-performance liquid chromatography system (Supplemental Figure 1).

#### Relationship to FeNO and eosinophils.

BAL fluid GSSG and GSH:GSSG correlated with FeNO, indirectly suggesting T2 inflammation may interact with airway redox biology (Table 2 and Figure 1C). As for BAL fluid, intracellular GSH and GSH:GSSG negatively correlated with FeNO, again supportive of a contribution from T2 inflammatory processes (Table 2, Figure 1C, and refs. 11, 12). However, neither BAL fluid nor intracellular GSH, GSSG, and GSH:GSSG correlated with BAL eosinophils or any other inflammatory cell, suggesting eosinophilia plays a lesser role in contributing to higher extracellular/BAL fluid GSSG than epithelial cells do (Supplemental Tables 3 and 4).

### IL-13 upregulates 15LO1 pathway and lowers intracellular GSH

IL-13 increases the expression and activity of 15LO1, while inducing binding to PEBP1 (6, 7, 10, 13). This binding switches 15LO1’s preferred substrate from free arachidonic acid to arachidonic acid conjugated to PE, which generates ferroptotic lipids, including the hydroperoxy-phospholipid 15-hydroperoxy-eicosatetraenoic acid–PE (15-HpETE-PE) (6, 7, 10, 13).

**Figure 2:**
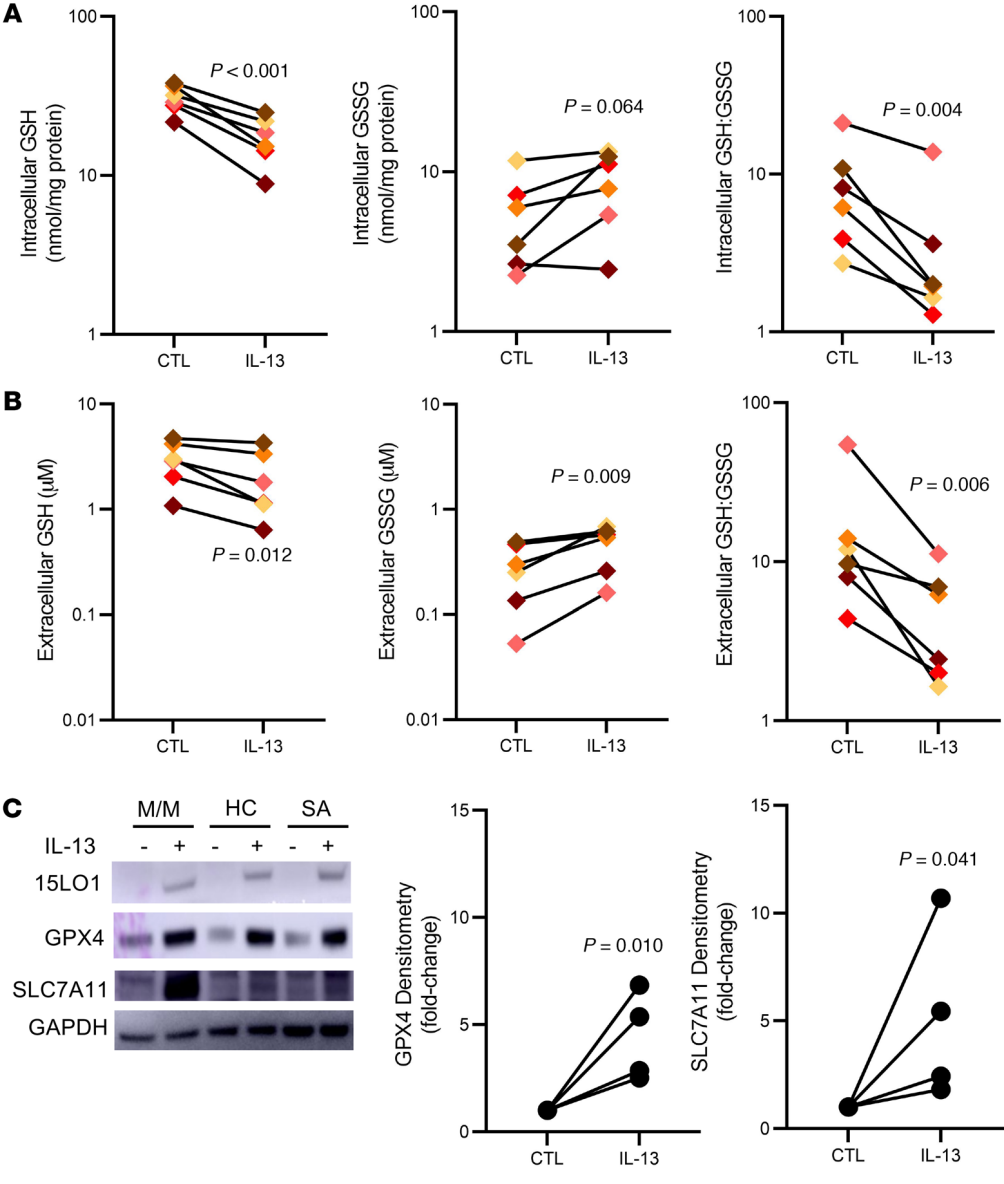
IL-13 lowers alters the intra- and extracellular oxidative potential by decreasing intracellular GSH and GSH:GSSG and increasing extracellular GSSG. (**A**) IL-13 decreases intracellular GSH levels and lowers the GSH:GSSG ratio (*n =* 6). (**B**) IL-13 also decreases extracellular GSH levels but increases extracellular GSSG, lowering GSH:GSSG (*n =* 6). (**C**) IL-13 (10 ng/mL) increases expression of 15LO1, GPX4, and SLC7A11 by Western blot, with GPX4 and SLC7A11 increases quantified by densitometry (*n =* 4–12). Matched-pair analysis of log-transformed data was used to compare conditions.

Under homeostatic conditions, 15-HpETE-PE is rapidly and specifically metabolized by the GSH-dependent GPX4 to the nonreactive hydroxy derivative 15-HETE-PE (6, 14). This activity could consume GSH and lower intracellular levels of reduced intracellular GSH. Direct oxidation of GSH to GSH• through free radical intermediates formed from 15-HpETE-PE before it is metabolized could also occur. In line with these metabolic possibilities, we observed that previously described increases in 15LO1 pathway proteins and activity were accompanied by decreases in both intracellular GSH (*P <* 0.001) and GSH:GSSG (*P =* 0.004; Figure 2A). Although there were only marginal increases in intracellular GSSG levels (*P =* 0.064), IL-13 increased extracellular (apical supernatant) GSSG (*P =* 0.009), while lowering both GSH (*P =* 0.012) and GSH:GSSG (*P =* 0.006; Figure 2B), supportive of intracellular compensatory mechanisms that mirror the ex vivo (BAL and fresh HAEC) findings. In this regard, as 15LO1 increased, GPX4 expression concomitantly increased to balance the high 15LO1 activity (Figure 2C). In addition, SLC7A11, the antiporter providing intracellular cystine (necessary to maintain intracellular GSH levels) also increased (Figure 2C and Supplemental Figure 2A). These data support an effect of IL-13 to decrease total and reduced GSH through high 15LO1, PEBP1, and GPX4 activity, which is partially compensated by (a) increased expression/activity of SLC7A11 to enhance new intracellular GSH production and (b) increased export of GSSG.

### 15LO1 increases intracellular oxidative potential by lowering GSH and increasing extracellular GSSG

**Figure 3:**
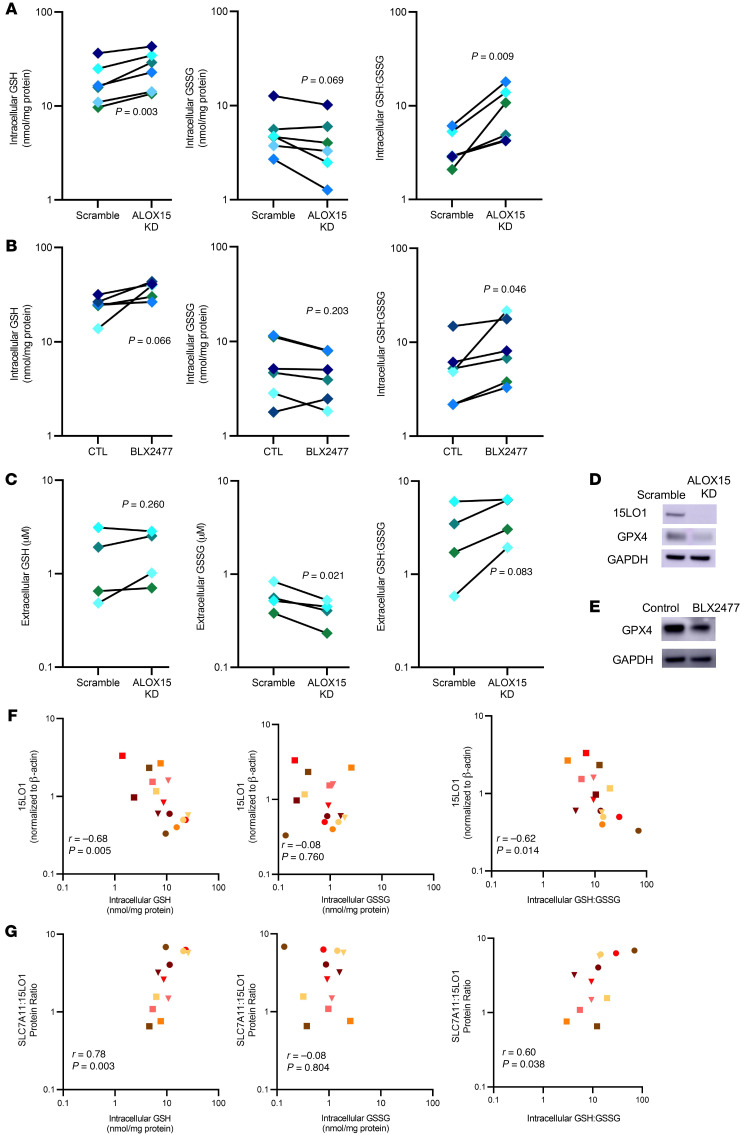
15LO1 expression and activity regulate intracellular redox balance by lowering GSH and GSH:GSSG. (**A**) Under IL-13–stimulated conditions, *ALOX15* (15LO1) knockdown (KD) increases intracellular GSH and the GSH:GSSG ratio toward control conditions (*n =* 6). (**B**) Under IL-13 conditions, inhibition of 15LO1 by the specific 15LO1 inhibitor BLX2477 also restores intracellular GSH and GSH:GSSG (*n =* 6). (**C**) *ALOX15* KD lowers extracellular GSSG and restores a normal GSH:GSSG ratio (*n =* 6). (**D**) Representative Western blots demonstrating that *ALOX15* KD also decreases GPX4 protein expression (*n =* 4). (**E**) Representative Western blots show decreased expression of GPX4 under conditions of 15LO1 inhibition (*n =* 4). (**F**) Ex vivo 15LO1 protein expression correlates with intraepithelial cell GSH and GSH:GSSG (*n =* 15). (**G**) Indexing SLC7A11, as a critical regulator of intracellular GSH levels, to 15LO1 improves the relationship of 15LO1 to intracellular GSH (*n =* 12). Matched-pair analysis (**A**–**C**) of log-transformed data was used to compare conditions, while Pearson’s correlation was used for **F** and **G**.

To confirm that 15LO1-PEBP1 complex activity alters IL-13–induced intracellular GSH levels, we modeled these relationships in vitro by stimulating cultured HAECs (at ALI) with IL-13 for 7–10 days in the presence and absence of 15LO1 protein (dicer siRNA knockdown [KD] of *ALOX15* gene) or enzymatic activity (specific 15LO1 inhibitor BLX2477) (14, 15). *ALOX15* KD normalized the intracellular GSH (*P =* 0.003), while chemical inhibition of 15LO1 had a marginal, but insignificant effect (*P =* 0.066). GSH:GSSG increased (both *P <* 0.05) to levels seen in control wells. Intracellular GSSG was marginally decreased by KD of *ALOX15* (*P =* 0.069), but not by chemical inhibition (*P =* 0.203; Figure 3, A and B). These small differences in efficacy of BLX2477 may be explained by the 3-log higher IC_50_ of BLX2477 for free versus PE-esterified eicosatetraenoic acid (ETE) (Supplemental Figure 3). *ALOX15* KD did, however, lead to decreased extracellular GSSG (*P =* 0.021), with marginal but insignificant increases in extracellular GSH:GSSG (*P =* 0.083). It did not impact extracellular GSH (*P =* 0.260; Figure 3C). Of note, KD of *ALOX15* and inhibition of 15LO1 decreased GPX4 protein expression, consistent with less need for peroxidase activity. There were inconsistent and nonsignificant reductions in SLC7A11 (Figure 3, D and E, and Supplemental Figure 2, B and C).

We previously reported that IL-13 increases intracellular 15-HpETE-PE via 15LO1 in vitro in modest molar amounts (see also Supplemental Figure 4A and ref. 7). These picomolar levels of 15-HpETE-PE alone (Supplemental Figure 4A, generated under IL-13 plus arachidonic acid conditions) would be insufficient to directly oxidize the nanomolar changes caused by oxidation of GSH to GSSG observed in response to IL-13 (or the change with inhibition of 15LO1 or KD of *ALOX15*/15LO1). Given that GPX4 can reduce hydroperoxy derivatives of different lipids, we analyzed the amounts of all major hydroperoxy- and hydroxy-phospholipids impacted by IL-13 by LC-MS/MS (see Methods). As seen in Supplemental Figure 4B, the sum of total intracellular hydroperoxy- and hydroxy-phospholipids following IL-13 stimulation was approximately 2 nmol/mg protein, that is, sufficient amounts to quantifiably impact the change in intracellular GSH (and the increases in GSSG) under these two conditions, as shown in Supplemental Figure 4C under similar conditions. Thus, the loss of intracellular GSH in the presence of active generation of hydroperoxy-phospholipids could occur through several active mechanisms, including formation of GSH•, export of GSSG, inadequate replenishment of GSH, or insufficient levels of GSSG reductase, each directly or indirectly dependent on the generation of hydroperoxy-phospholipids. It should also be noted that the actual amounts of hydroperoxy-phospholipids formed after IL-13 treatment may be even greater if one accounts for the electrophilic secondary products of hydroperoxy-phospholipid decay that attack the nucleophilic sites of proteins and form covalent adducts.

These in vitro mechanisms were recapitulated ex vivo, as observed by the strong correlations of ex vivo 15LO1 protein with intracellular GSH (*r* = –0.68, *P =* 0.005) and GSH:GSSG (*r* = –0.62, *P =* 0.014) in freshly brushed HAECs (Figure 3F). Because SLC7A11 regulates GSH synthesis (through intracellular cystine availability) and 15LO1 decreases intracellular GSH, a high SLC7A11 to 15LO1 protein ratio likely supports high/normal intracellular GSH. Indeed, intracellular GSH (*r* = 0.78, *P =* 0.003) and GSH:GSSG (*r* = 0.60, *P =* 0.038) positively correlated with the SLC7A11/15LO1 protein ratio, without relation to intracellular GSSG. This suggests that under T2 “stressed” conditions, increases in SLC7A11 activity help maintain intracellular redox balance (Figure 3G). Thus, we identify 15LO1 pathway activity in vitro as a prime driver of lower intracellular reduced GSH levels and export of GSSG. Under steady-state conditions, this GSSG is rapidly exported to extracellular fluid, potentially contributing to the higher levels of GSSG measured in asthmatic BAL fluid, while also contributing to an altered intracellular redox balance.

### Further alterations in intracellular redox balance driven by lower GSH availability induce cell death and enhance T2 signature protein expression

**Figure 4:**
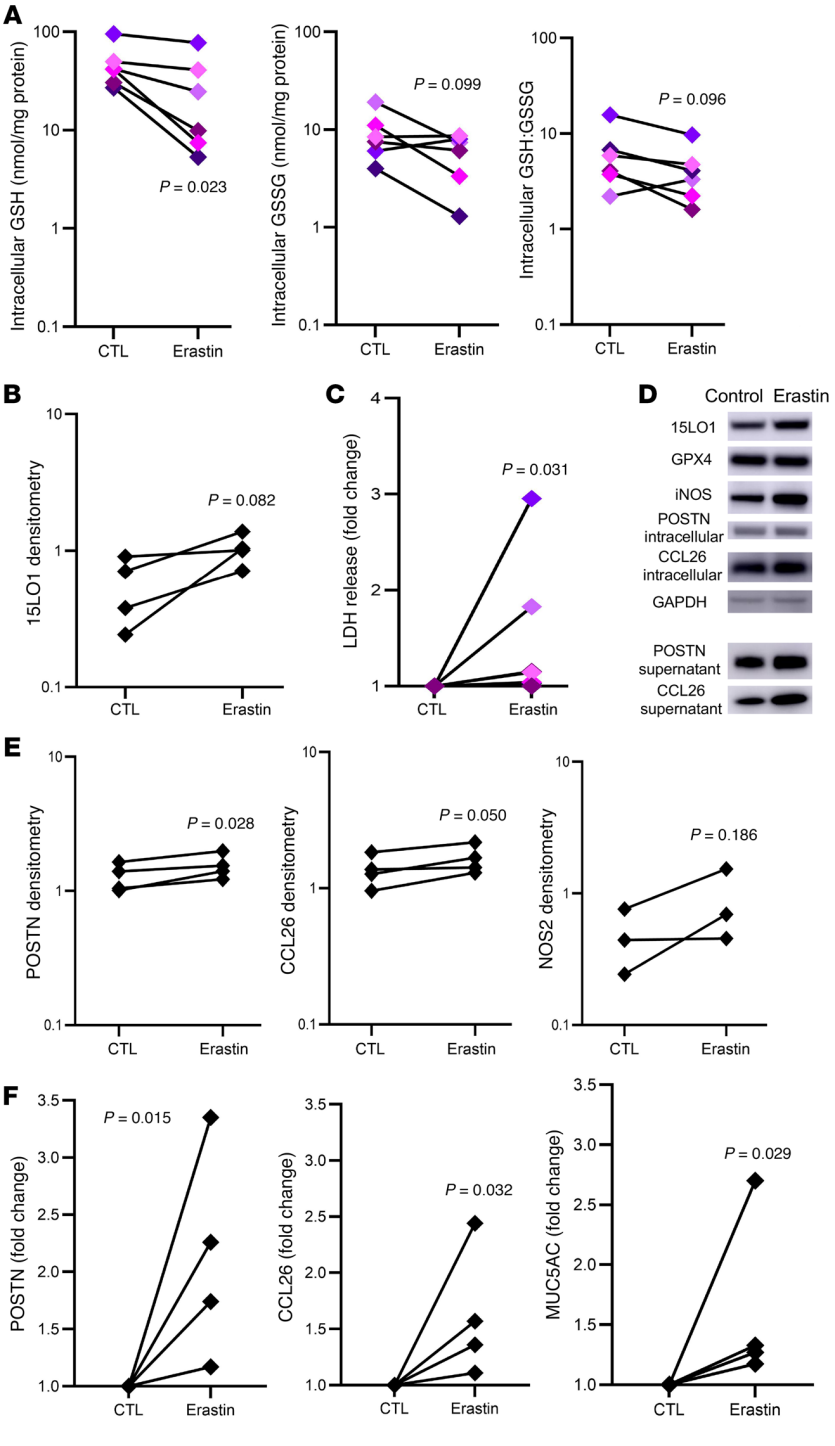
Activity of the cystine-glutamate antiporter SLC7A11 modulates intracellular redox, cell death, and type 2 signature protein expression. (**A**) Inhibition of SLC7A11 activity by erastin lowers intracellular GSH and the GSH:GSSG ratio (*n =* 6). (**B**) This decrease in GSH:GSSG is accompanied by increased expression of 15LO1 (*n =* 4). (**C**) The decreased GSH:GSSG increases LDH release, consistent with activation of oxidatively driven cell death (*n =* 6). (**D**) Representative Western blot of intracellular and secreted type 2 signature markers following SLC7A11 chemical inhibition with erastin for 24 hours (*n =* 3–4). (**E**) Densitometry results showing increases in intracellular periostin (POSTN), CCL26, and iNOS following treatment with erastin (*n =* 3–4). (**F**) Densitometry results showing parallel fold-change increases in basilar secretion of POSTN and CCL26, with increased apical secretion of MUC5AC protein following treatment with erastin (*n =* 4). Matched-pair analysis following log transformation was used to compare the conditions.

We previously demonstrated that 15LO1-PEBP1 complex activation consistently upregulates the expression of T2-signature gene/protein expression, including MUC5AC, periostin (POSTN), and CCL26 (6, 10, 14). Although the mechanism for this upregulation is not fully characterized, altered redox states are known to enhance expression of numerous genes/proteins. To determine whether increases in intracellular GSH directly enhance expression of T2-signature proteins in HAECs, we lowered intracellular GSH levels by inhibiting SLC7A11 using the chemical inhibitor erastin (16). As anticipated, chemical inhibition of SLC7A11 by erastin lowered intracellular GSH and GSH:GSSG (Figure 4A). Interestingly, these changes were accompanied by marginal increases in 15LO1 (*P =* 0.082), potentially further contributing to GSH oxidation (Figure 4B). Physiologically, this lowering of GSH led to increased LDH release (*P =* 0.031), consistent with redox activation of ferroptosis and cell death (Figure 4C and refs. 8, 17). Worsening intracellular oxidative potential was accompanied by increases in intracellular expression of downstream T2 signature proteins, such as POSTN and CCL26, with nonsignificant increases in inducible NO synthase (iNOS/NOS2; Figure 4, D and E). In addition to enhancement of intracellular expression, basal secretion of POSTN and CCL26 increased in parallel (Figure 4, D and F). iNOS is not a secreted protein and was not evaluated for secretion. Apical protein secretion of MUC5AC (by ELISA; Figure 4F) was also increased with erastin, as compared with IL-13 alone. These results confirm that decreases in intracellular GSH (and associated increased oxidative stress) worsen T2 inflammatory protein expression and secretion in HAECs, while increasing their susceptibility to ferroptotic death.

### BAL fluid and intracellular GSH pathways correlate with HAEC T2-associated gene expression

To determine whether these in vitro findings were reflected ex vivo, BAL fluid GSSG levels were compared to fresh HAEC expression of T2-related genes from 2 different RNA-Seq databases (SARP3 and IMSA), which were harmonized and merged as outlined in the Methods (Table 3). BAL fluid GSSG strongly and positively correlated with *ALOX15* expression, with weaker relationships to *POSTN* and *NOS2* expression. Importantly, *ALOX15* expression also correlated strongly with a T2 inflammatory gene signature (Supplemental Table 5), supporting the link between the GSH pathway, T2 inflammation, and 15LO1. Perhaps not surprisingly, intracellular GSH levels more strongly correlated with fresh HAEC expression of *ALOX15* (Table 3). These results support the hypothesis that the T2-induced 15LO1 pathway–induced alteration of intracellular redox balance enhances T2 inflammatory responses ex vivo.

### Altered redox balance ex vivo correlates with worsened clinical and physiologic outcomes

#### BAL fluid.

**Figure 5:**
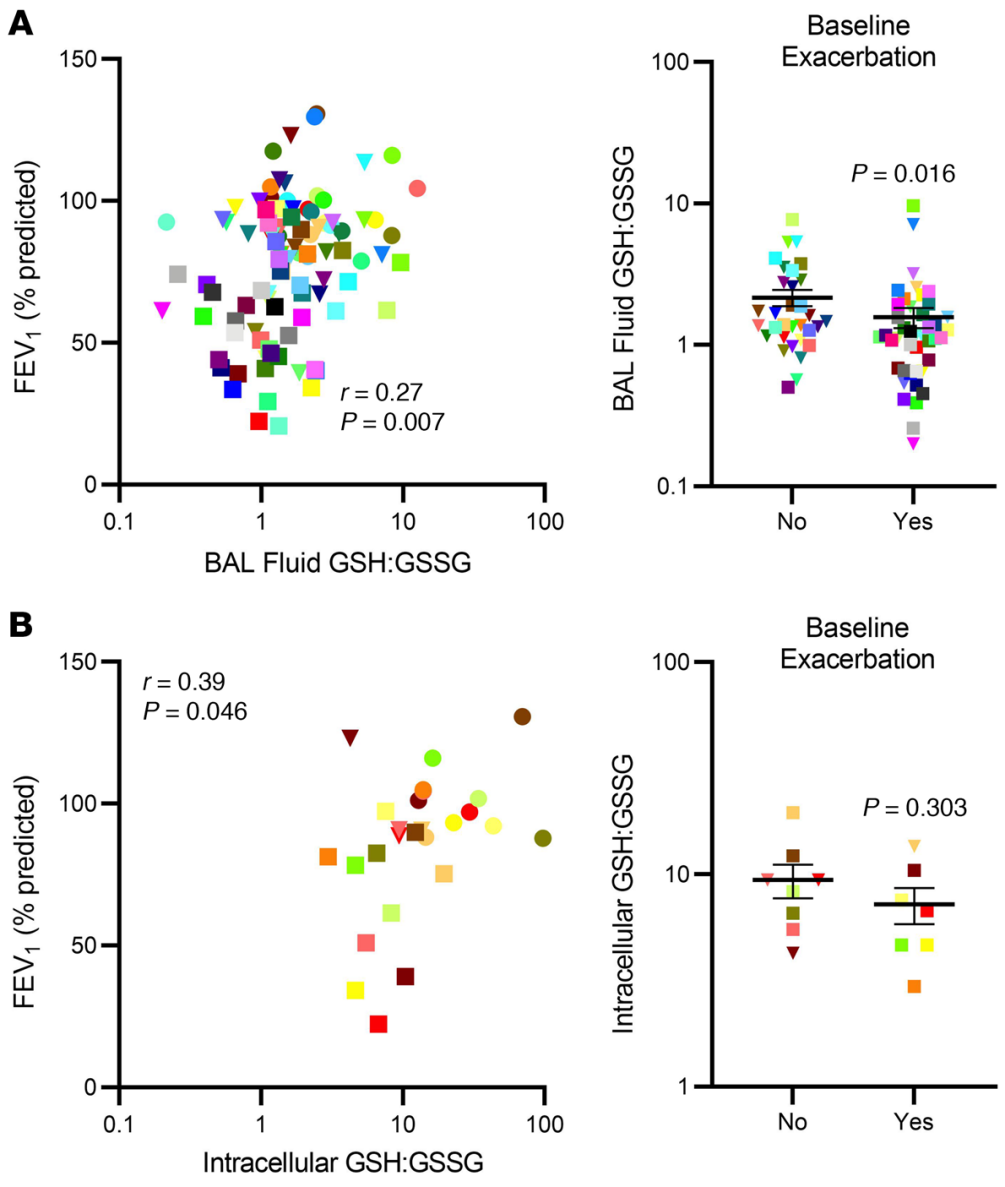
Ex vivo extra- and intracellular redox balance is associated with clinical outcomes relevant to asthma. (**A**) Extracellular BAL fluid GSH:GSSG ratio correlates with lung function (*n =* 98), as measured by predicted FEV_1_% (*n =* 75) and associates with asthma exacerbations. (**B**) Intracellular GSH:GSSG correlates with predicted FEV_1_% (*n =* 26), while in small numbers the association with baseline exacerbations (*n =* 15) is not significant. Pearson’s correlation testing was performed for comparisons of predicted FEV_1_% with GSH:GSSG, while *t* tests were used for group comparisons. Data were log transformed except for predicted FEV_1_%, which was normally distributed. Bars represent mean and error bars represent SEM.

Both BAL fluid GSSG and GSH:GSSG were (differentially) correlated with FEV_1_ (Table 2 and Figure 5A). Similarly, patients who experienced asthma exacerbations in the previous year had lower BAL fluid GSH:GSSG than those who did not (Figure 5A). Asthma Quality of Life Questionnaire (AQLQ) score correlated with BAL fluid GSH:GSSG as well (*r* = 0.33, *P =* 0.005). These data suggest a relationship of worsening airway obstruction and asthma control with higher GSH-related oxidative stress measures.

#### Intracellular.

Intracellular GSH:GSSG also correlated with FEV_1_ (% predicted), with a numerically stronger correlation than observed in BAL fluid (Figure 5B). Intracellular GSH also positively correlated with AQLQ score (Table 2). Intracellular GSH:GSSG did not differ by history of asthma exacerbations, perhaps due to the smaller sample size (Figure 5B).

## Discussion

Variation in redox state, as represented by the intracellular GSH:GSSG ratio, can have profound clinical implications, enhancing cell signalling and even cell death. The studies outlined here identify a specific pro-oxidant pathway that dramatically impacts both intra- and extracellular redox states, enhances T2 inflammation and cell death, and is associated with worsening asthma control. Almost all inflammatory diseases exhibit evidence of enhanced oxidative stress (18). Yet, treatment of disease with antioxidant approaches has nearly uniformly failed (19, 20), perhaps partly because oxidative stress is often compartmentalized or driven by multiple nonspecific (chemical) and specific (enzymatic) pathways. Here, we confirm previously reported evidence of redox abnormalities in asthma (3), but greatly expand on these findings to identify a specific intracellular mechanism regulating these levels. We identify high HAEC 15LO1 activity in vitro as a prime driver of worsening (lower) intracellular (epithelial) GSH:GSSG observed in association with T2-high asthma ex vivo. Loss of intracellular GSH is likely multifactorial and includes generation of high levels of hydroperoxy-phospholipids (which directly or indirectly oxidize GSH and limit GSSG recycling by reductases) as well as active export of GSSG generated by high GPX4 activity (7, 8, 21). In this setting, any perturbation that lowers intracellular GSH to an as yet unknown threshold could eventually induce cell death and, in all likelihood, ferroptosis, but also significantly increase expression and secretion of numerous T2-associated proteins.

A consistent imbalance in GSH and GSSG has been observed in asthma; however, the mechanisms for this imbalance have been unclear (22). Although studies have suggested a role for superoxide dismutase (23), the relationships with redox changes and clinical parameters have generally been weak, supporting involvement of other pathways. However, no studies to date have reported intracellular GSH levels in relation to asthma or specific lower airway cell type. Epithelial cells, the lining cells of the lung, by both position and abundance, are likely to contribute substantially to BAL fluid GSH:GSSG, through increasing export of GSSG under conditions of endogenous (or exogenous) redox changes. While significantly lower GSH:GSSG ratios were not seen using traditionally defined severity phenotypes, lower ratios corresponded to a greater history of exacerbations, lower lung function, and higher FeNO. In line with the intracellular contributions to these clinical findings, correlation of intraepithelial cell GSH levels to FEV_1_ was considerably stronger than the relationship in BAL fluid, supporting the overall importance of endogenous epithelial cell responses to clinical outcomes.

Ferroptosis is strikingly driven by worsening redox imbalance, which generates and maintains high levels of hydroperoxy-phospholipids (7, 24). In IL-13–stimulated HAECs, it is specifically driven by 15LO1-PEBP1 complex–catalyzed generation of 15-HpETE-PE (7). Under steady-state conditions, these hydroperoxy-phospholipids are metabolized to hydroxy-phospholipids by GPX4, consuming large quantities of GSH and generating GSSG (25). Normally, this GSSG is recycled by reductases to maintain sufficient intracellular levels of reduced GSH. However, these events are not without redox consequences, as shown here. Under conditions of IL-13 stimulation, intracellular GSH levels (and hence GSH:GSSG) fall through 15LO1- and PEBP1-dependent reactions. This loss of GSH occurs through several mechanisms, including direct enzymatic GSH oxidation to GSSG by GPX4 during reduction of hydroperoxy-phospholipids, export of GSSG, and dependence on the continuous availability of newly formed GSH. It is also possible that radical intermediates nonenzymatically produced from hydroperoxy-phospholipids directly react with GSH, leading to the formation of a highly pro-oxidant GS• radical. Finally, GSH could also be consumed under conditions of oxidative stress by a process known as *S*-glutathionylation (26). This process was reported to also be active in epithelial cells under T2 conditions, in association with 15LO1 (27). However, its relative impact on overall intraepithelial cell levels of GSH, or activation of other proinflammatory mediators, is unclear and requires further study. When GSH regeneration is further limited by inhibition of SLC7A11 (with erastin), cell death ensues. Our data suggest activation of ferroptotic pathways could be one of the primary drivers of intracellular (and extracellular) redox imbalance in T2-high asthma.

Previous reports from our laboratory showed that 15LO1 also contributes to downstream T2 immunoinflammatory responses (6, 10, 14, 28). While multiple mechanisms may exist for these increases, it is widely appreciated that increased oxidative potential enhances receptor-mediated downstream responses. Although ligand-receptor pathways are often thought of as linear paths from engagement to transcription/translation, many studies show that enhanced oxidative potential can augment or suppress signaling pathways, including those related to MUC5AC, while more recently, oxidatively driven epigenetic effects on gene transcription have also been reported (29–32). However, given the lower intracellular GSH:GSSG ratio after IL-13 stimulation, it is difficult to determine the proportionate role that loss of GSH plays in downstream T2-pathway mRNA/protein expression. This is especially difficult because under IL-13 conditions, upregulation of additional factors, including GPX4 and SLC7A11 (9), occur in an effort to maintain intracellular homeostasis. Similarly, increased export of GSSG also occurred, potentially contributing to lower intracellular levels. Although the mechanisms are unclear, multidrug resistance protein 1 (otherwise known as ABCC1) has been reported to preferably export GSSG under conditions of high intracellular GSSG (33, 34).

Any immune or inflammatory settings in which the amount or activity of any of these proteins is lowered or does not increase appropriately (as we modeled with erastin) could dramatically perturb homeostatic balance and enhance T2 inflammatory factor expression and oxidatively driven cell death. In fact, worsening asthma control and exacerbations are consistently linked to lower lung GSH levels and higher oxidative potential (35, 36). Exogenous factors, including various pollutants, could further increase extracellular GSSG, perhaps limiting the export of GSSG against a concentration gradient. Endogenously, interferon γ lowers SLC7A11 activity (37), suggesting that viruses, which activate interferons of all types, could alter this fragile intracellular GSH homeostasis. This, in turn, would drive oxidatively induced cell death and increase barrier dysfunction and T2 inflammation, potentially identifying a mechanism by which viruses cause asthma exacerbations. Although not evaluated here, lower intracellular GSH levels would also deactivate the GSH-dependent GPX4, further enhancing ferroptotic cell death (8, 38). These factors could become self-perpetuating as more oxidative stress is generated by the inflammatory responses, promoting persistence of disease.

Our study has some limitations. GPX4 activity (levels of 15-HpETE-PE and 15-HETE-PE) was not directly measured in relation to inflammatory marker expression and cell death. However, we previously reported intracellular levels of these mediators in relation to GPX4 activity (7) that are likely similarly altered here. We chose to focus on SLC7A11 as playing a critical role in intracellular GSH levels, targeting SLC7A11 with erastin. Multiple other transporters and enzymes, including the availability of GSSG reductase, thioredoxins, and activity of glutathione transferases are also involved in maintenance of the intracellular GSH-GSSG balance, which could be evaluated in the future. Although our fresh epithelial brushing cell GSH data could have been influenced by non–epithelial (inflammatory) cell contributions, the percentages of these cell types in the brushings are typically less than 10% of total cells, and there was no correlation of BAL inflammatory cells and BAL GSH or GSSG. However, a more detailed evaluation of inflammatory cells and activity in tissues or epithelial brushings is needed before their contribution can be ruled out. Finally, further work is required to understand the mechanisms by which increased oxidative potential leads to increased T2-signature protein levels.

In conclusion, these findings strongly suggest that the altered airway redox imbalance consistently reported in asthma is primarily driven by high T2-associated 15LO1 activity in HAECs. This redox imbalance promotes enhanced expression of T2-associated proteins, leaving the cells vulnerable to oxidatively induced cell death and, potentially, to asthma exacerbations. The 15LO1/PEBP1 pathway should be investigated as a novel asthma therapeutic target.

## Methods

### Subjects.

Subjects were enrolled in SARP or IMSA studies between February 2013 and January 2019, as previously described (39). All SARP participants were from the University of Pittsburgh site. Participants were never smokers, or had smoked less than 5 pack-years, with no smoking in the last year. HCs had no history of respiratory disease or recent respiratory tract infection and normal pulmonary function. All asthma patients met American Thoracic Society (ATS) criteria of asthma (40). Patients were classified as severe asthma defined by 2014 European Respiratory Society/ATS guidelines (41). BAL fluid measurements from 2 different cohorts (SARP and IMSA) were combined to evaluate the GSH pathway.

### Clinical and physiologic data.

Prior to bronchoscopy, subjects underwent an extensive workup that included medical history, asthma control questionnaires, physical examination, FeNO concentration measurements, pulmonary function tests, and blood tests. Patients with exacerbation were identified from self-reported use of systemic corticosteroid courses lasting 3 or more days for asthma in the past 12 months on visit 1. Questions regarding hospitalization for asthma were also reviewed. Asthma control was assessed with the ATS AQLQ. FeNO was measured before bronchodilator administration at a constant exhalation flow rate of 50 mL/s with a NIOX analyzer, according to ATS recommendations (42). Baseline pulmonary function tests were performed with standard medication withholds.

### Bronchoscopy.

Bronchoscopy with epithelial brushings and BAL were performed as previously described (6, 43, 44).

### ALI cell culture system and dicer siRNA.

After proliferation submerged culture condition, the first-passage HAECs were seeded in 12-well Transwell plates as previously described (39, 45). When confluent, HAECs were shifted to ALI culture with 50 μL apical media for 8 days to achieve full differentiation with and without IL-13 (10 ng/mL) stimulation (R&D Systems, 213-ILB).

Dicer siRNA KD of *ALOX15* was performed in 12-well Transwell plates, as previously described (45). After transfection for 24 hours, IL-13 was added into lower chamber media every 48 hours for up to 7 days. *ALOX15* dicer siRNA was purchased from IDT.

A selective enzymatic inhibitor of 15LO1, BLX2477 (gift from Hans-Erik Claesson, Karolinska Institutet, Stockholm, Sweden; ref. 15), was added at a final concentration of 2 mM for 24 hours. A selective enzyme inhibitor of SLC7A11, erastin (Sigma-Aldrich, E7781; ref. 19) was added at 10 μM into the medium for 24 hours before harvest, with dimethyl sulfoxide or water added as vehicle control. Total protein from freshly brushed and cultured HAECs was collected in cell lysis buffer, homogenized by sonication, and then centrifuged at 1,699*g* for 10 minutes. The apical supernatant was used for GSH:GSSG and protein analysis.

### RNA-Seq data analysis of bronchoscopy brushings.

RNA-Seq data from 2 different cohorts (SARP and IMSA [GSE 158752]) were analyzed. The RNA-Seq data for SARP are deposited in NCBI’s dbGaP (accession numbers phs001119.v1.p1 and phs001446, respectively). While awaiting data release via dbGaP, investigators may contact the senior author (Wenzel) of this study or go to the SARP website (46) to access to the data via the ancillary study mechanism for SARP. Count data for the SARP cohort was obtained from Stephanie Christianson (UCSF) and as published recently (47).

To ensure harmonization with the SARP data set, the count data for the IMSA cohort was generated using the same processing pipeline that was used to generate the SARP count data; reads were trimmed using TrimGalore (48) and aligned to Ensemble hg38 using STAR (49). Counts for known genes were then generated using featureCounts (50).

After obtaining count data for the IMSA cohort, the IMSA and SARP count data were processed together. The count data were filtered to remove genes with low expression, transformed using the variance stabilizing transformation from the DESeq2 package (51), and then batch corrected using ComBat (52). We found that processing the counts from the 2 cohorts together and correcting for batch effects with ComBat effectively harmonized the data from the 2 cohorts. This harmonized data set was then used for the RNA-Seq data analysis (Supplemental Table 5).

### GSH and GSSG measurement.

GSH and GSSG levels were measured as previously described (21). GSH and GSSG levels were determined as the difference in low molecular weight thiols. BAL fluid, cell lysates, or apical culture supernatants were incubated with glutathione peroxidase (Sigma-Aldrich, G6137), cumene hydroperoxide (Sigma-Aldrich, 247502), glutathione reductase (Sigma-Aldrich, G9297), and NADPH (Sigma-Aldrich, N1630) for 30 minutes at 25°C. Thiol Fluorescent Probe IV (10 mM; Sigma-Aldrich, 1173888-41-9) was then used to identify the low molecular weight thiols, with fluorescence measured at 400 nm excitation and 465 nm emission. A standard curve was established by the addition of GSH to PBS. Intracellular GSH and GSSG levels were corrected for intracellular protein, determined by Pierce BCA Protein Assay Kit (Thermo Fisher Scientific, 23225) using albumin as a standard. The lower limit of detection for this assay was 0.016 μM or 0.13 nmol/mg protein.

### High-performance liquid chromatography analysis of low molecular mass thiols and disulfides.

Quantification of GSH in BAL fluid was performed as reported by Steele et al. (53). Briefly, GSH was derivatized with 4-fluoro-7-aminosulfonylbenzofurazan (ABD-F) to the fluorescent GS-ABD analyte. After separation on a C18 reverse-phase high-performance liquid chromatography column (Beckman, 4.6 × 150 mm; particle size, 5 μ), GS-ABD was detected with a Waters 474 scanning fluorescence detector [λ(ex) = 390 nm; λ(em) = 530 nm]. Authentic GSH was used as an external standard. GSSG in the samples was reduced with tris(2-carboxyethyl)phosphine (TCP) to GSH, and then derivatized with ABD-F to obtain the total concentration of thiols ([GSSG] = [GSH]_total_ – [GSH, prior to reduction with TCP]).

### LC-MS/MS analysis of oxygenated phospholipids.

Lipids were extracted using the Folch procedure, and phosphorus was determined by a micromethod as described previously (7, 21). Phospholipids and their hydroxy and hydroperoxy derivatives, including 15-HETE-PE and 15-HpETE-PE, were analyzed by LC/MS using a Dionex Ultimate 3000 HPLC system coupled on-line to an Orbitrap Fusion Lumos mass spectrometer (Thermo Fisher Scientific) using a normal phase column (Luna 3 μm silica C18(2) 100 Å, 150 × 2.0 mm, Phenomenex). The column was maintained at 35°C. The analysis was performed using gradient solvents (A and B) containing 10 mM ammonium formate at a flow rate of 0.2 mL/min. Solvent A contained isopropanol/hexane/water (285:215:5, v/v/v), and solvent B contained isopropanol/hexane/water (285:215:40, v/v/v). All solvents were LC/MS grade. The column was eluted for 0–23 minutes with a linear gradient from 10% to 32% B; 23 to 32 minutes with a linear gradient of 32% to 65% B; 32 to 35 minutes with a linear gradient of 65% to 100% B; 35 to 62 minutes held at 100% B; 62 to 64 minutes with a linear gradient from 100% to 10% B; followed by an equilibration from 64 to 80 minutes at 10% B. Analysis was performed in negative ion mode at a resolution of 140,000 for the full MS scan in a data-dependent mode. The scan range for MS analysis was *m*/*z* 400 to 1800 with a maximum injection time of 128 ms using 1 microscan. An isolation window of 1.0 Da was set for the MS scans. Capillary spray voltage was set at 3.5 kV, and capillary temperature was 320°C. The S-lens RF level was set to 60. Ion source conditions were set as follows: spray voltage = 4 kV, sheath gas = 20 (arbitrary unit), auxiliary gas = 4 (arbitrary unit), sweep gas = 0 (arbitrary units), transfer tube temperature = 300°C, RF-lens level = 50%. Analysis of raw LC/MS data was performed using software package Compound Discoverer 2.0 (Thermo Fisher Scientific) with an in-house-generated analysis workflow and oxidized-phospholipid database. Briefly, peaks with a signal to noise (S/N) ratio of greater than 3 were identified and searched against the oxidized-phospholipid database. Lipids were further filtered by retention time and confirmed by a fragmentation mass spectrum. Deuterated phospholipids (Avanti Polar Lipids) were used as internal standards. Values for *m*/*z* were matched within 5 ppm to identify the lipid species.

### Western blot.

Cell lysates and basilar supernatants were run in 4%–12% sodium dodecyl sulphate–polyacrylamide (SDS-PAGE) gels (Invitrogen) and transferred onto polyvinylidene difluoride membrane (Invitrogen). For supernatant studies, 40 μL of culture media was loaded as previously described (14).

Primary antibodies against SLC7A11 (Cell Signaling Technology, 12691, 1:500 dilution), iNOS (BD Biosciences, 610329, 1:500 dilution), 15LO1 (Abnova, H00000246-D01P, 1:1000 dilution), GPX4 (Abcam, 125066, 1:1000 dilution), POSTN (Santa Cruz Biotechnology, 67233, 1:500), and CCL26 (R&D Systems, AF653, 1:500 dilution) were used. GAPDH (Novus Biologicals, NB300-320, 1:1000 dilution) or β-actin (Sigma-Aldrich, A5441, 1:2000 dilution) was measured as control. Membranes were developed using an Amersham Imager 600 (GE Healthcare Life Sciences) with SuperSignal West Femto Maximum Sensitivity Substrate (Thermo Fisher Scientific, 34096). Densitometry analysis was performed using ImageJ software (NIH). See complete unedited blots in Supplemental Figure 5.

### MUC5AC and LDH measurements.

MUC5AC protein expression was quantified by ELISA (Novus Biologicals, NBP2-76703) and LDH by a Lactate Dehydrogenase Assay Kit (Abnova, KA0878) according to the manufacturers’ instructions. LDH was measured in both the culture medium and cell lysates, and LDH release (%) is expressed as (LDH levels in culture medium)/([LDH levels in culture medium] + [LDH levels in cell lysis]) × 100.

### Statistics.

All statistical analyses were performed with JMP Pro software version 14 (SAS Institute Inc.). Data were visualized with GraphPad Prism software version 8. For GSH-pathway measurements, each colored symbol represents an individual patient (circles = HC, triangles = M/MA, squares = SA) or individual human cell line, which can be followed through multiple figures. Most values were log transformed because they were linearly distributed; AQLQ scores were subjected to quantile normalization. Intergroup comparisons were performed by parametric *t* test and ANOVA, or χ^2^ tests. Cells from specific donors under 2 conditions were compared using matched-pair analysis. Pearson’s correlation was employed to analyze relationships among the continuous data. Bonferroni’s correction was applied to analyses correlating BAL fluid or intracellular GSH, GSSG, and GSH:GSSG to up to 5 gene expression variables or clinical parameters to adjust for multiple comparisons. A *P* value of less than 0.05 was considered statistically significant for univariate/1-group analyses. Any *P* value passing this significance (and correction) threshold is in bold text in the tables.

### Study approval.

The studies were approved by the University of Pittsburgh Institutional Review Board (IMSA, PRO15050456; local SARP, PRO12070359) and all subjects provided written informed consent prior to participation in the study and were identified by different numbers, not by names.

### Data and materials availability.

All data for this manuscript are included in the paper and raw data are available for review upon request.

## Author contributions

TN and SEW designed the study. JBT performed HAEC culture. TN, KY, YD, SNS, DS, YYT, JZ and VEK performed research studies. TN, AJS and SEW performed statistical analyses. SAC, SPG, PGW, JVF, AJS, and TN performed RNA-Seq analysis. TN, VEK and SEW interpreted the data. TN, AJS, AR, and SEW wrote the manuscript. All authors were involved in the preparation and review of the manuscript and approved the final submitted version.

## Supplementary Material

Supplemental data

## Figures and Tables

**Table 3:**
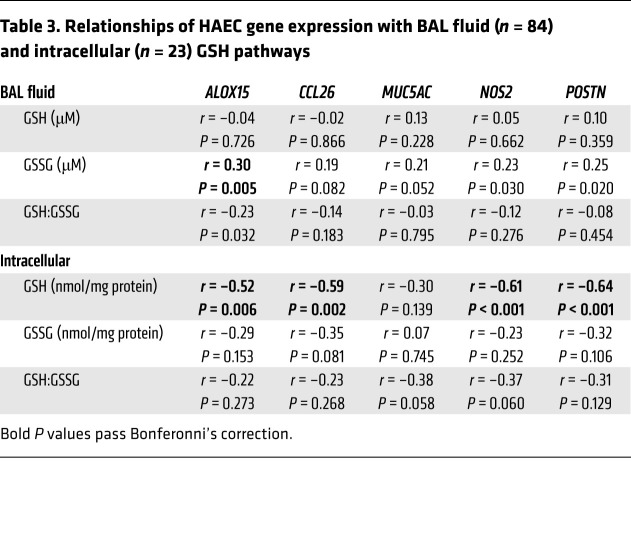
Relationships of HAEC gene expression with BAL fluid (*n* = 84) and intracellular (*n* = 23) GSH pathways

**Table 2:**
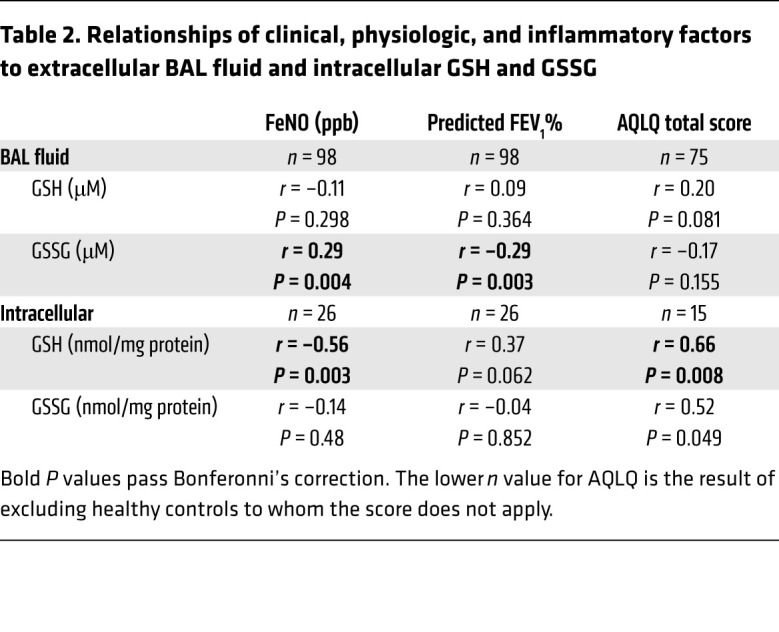
Relationships of clinical, physiologic, and inflammatory factors to extracellular BAL fluid and intracellular GSH and GSSG

**Table 1:**
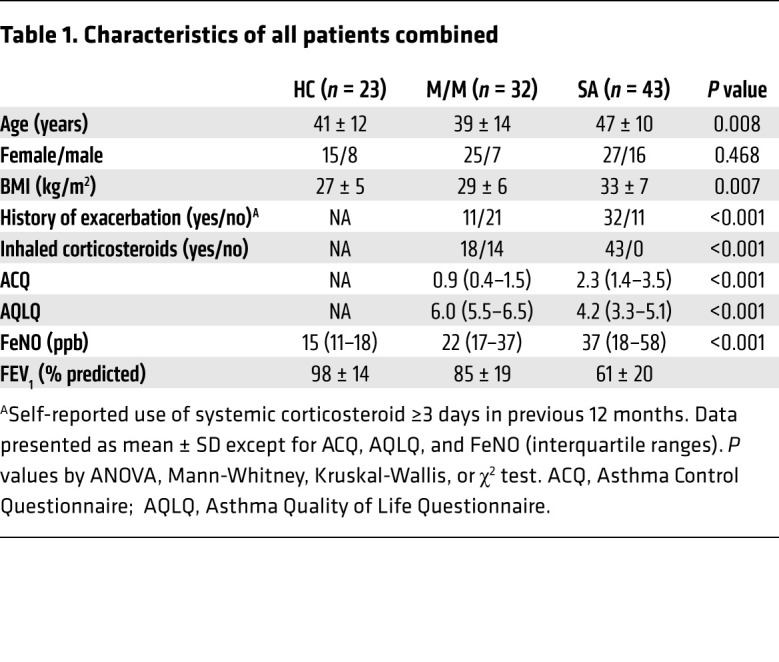
Characteristics of all patients combined

## Version 1 11/11/2021

In-Press Preview

## Version 2 01/04/2022

Electronic publication
