# Supplementary material for: 15LO1 dictates glutathione redox changes in asthmatic airway epithelium to worsen type 2 inflammation
Source: J Clin Invest. 2022 Jan 4;132(1):e151685. doi: 10.1172/JCI151685 (PMC8718153; doi:10.1172/JCI151685)
Supplement: Supplemental data [file jci-132-151685-s145.pdf]

透かしを削除する Wondershare PDFelement

カタログ

R2 supplementary Figures 1
R2 supplemental clean 5

Wondershare PDFelement

透かしを削除する Wondershare PDFelement

Supplemental Figure 1

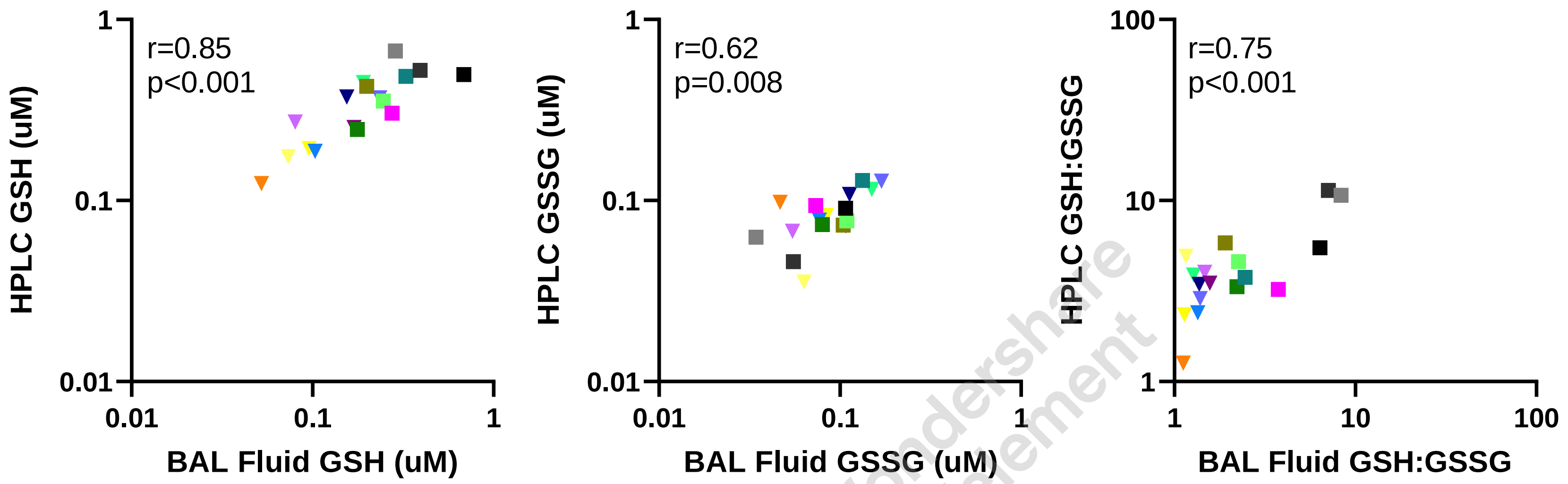

Wondershare PDFelement

Fig. 1. GSH and GSSG levels and GSH:GSSG measured by enzymatic method correlated strongly with high-performance liquid chromatography (HPLC) measurements (n=17).

透かしを削除する Wondershare PDFelement

Supplemental Figure 2.

A.

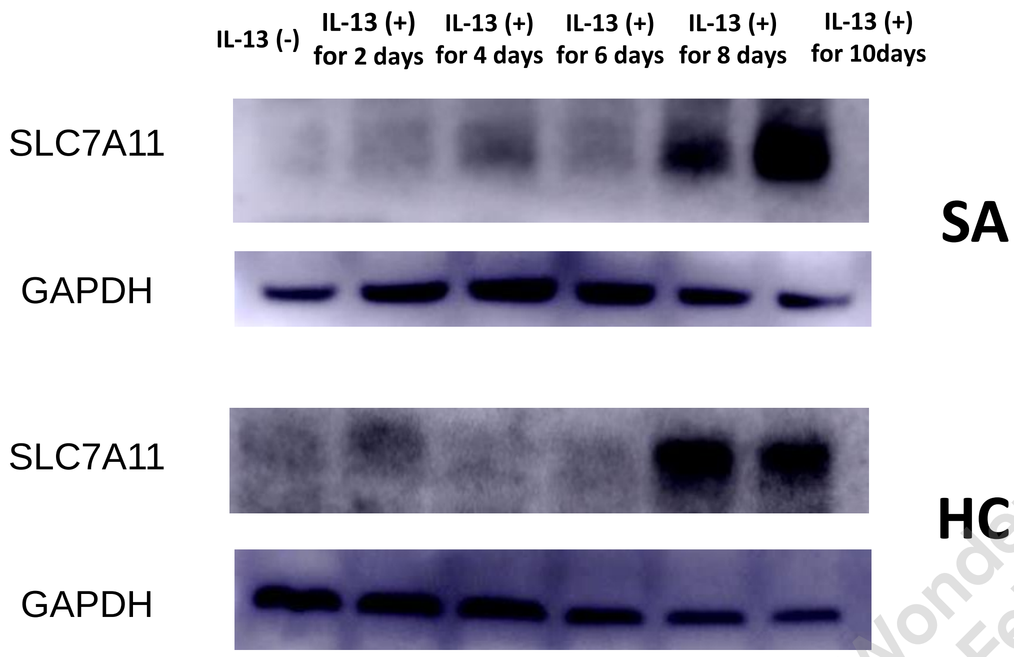

Fig. 2. (A) Representative western blots showing time course of SLC7A11 protein expression in response IL-13 stimulation *in vitro*. (**B**) Representative western bltos showing the inconsistent impact of ALOX15 siRNA knockdown on SLC7A11. (**C**) Densitometric analysis of the fold changes.

B.

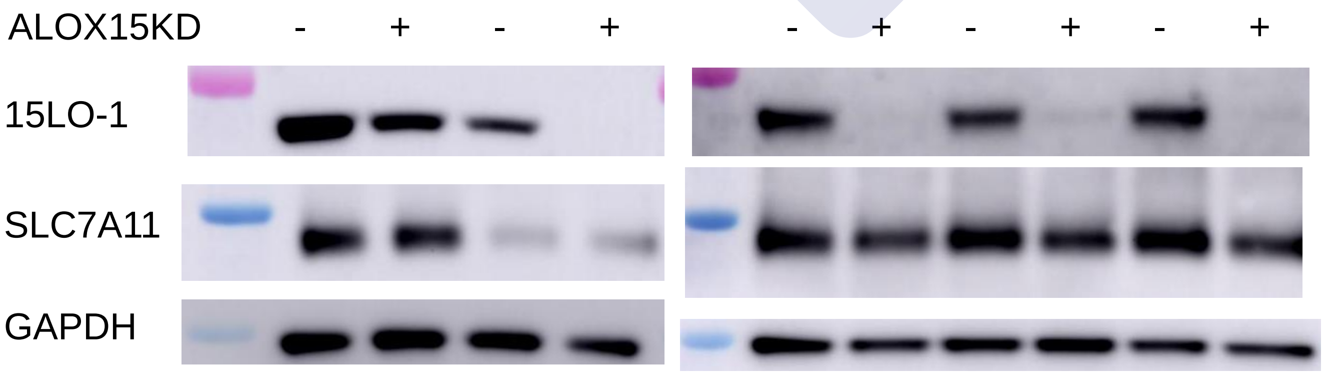

C.

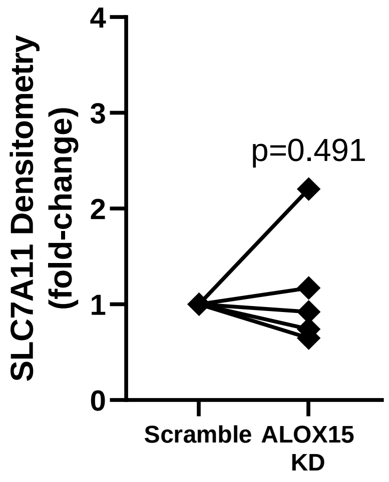

Wondershare PDFelement

透かしを削除する Wondershare PDFelement

Supplemental Figure 3.

A

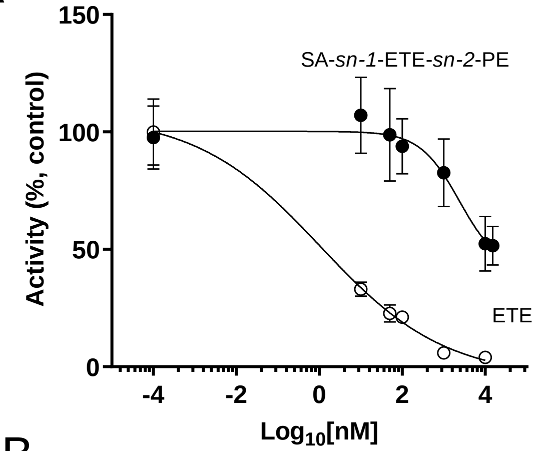

Sample	15-LOX-1 IC_{50} values
ETE	6.4 ± 0.2 nM
SA-sn-1-ETE-sn-2-PE	2.9 ± 0.4 μM
SA-sn-1-ETE-sn-2-PE+PEBP1	3.1 ± 0.5 μM

B

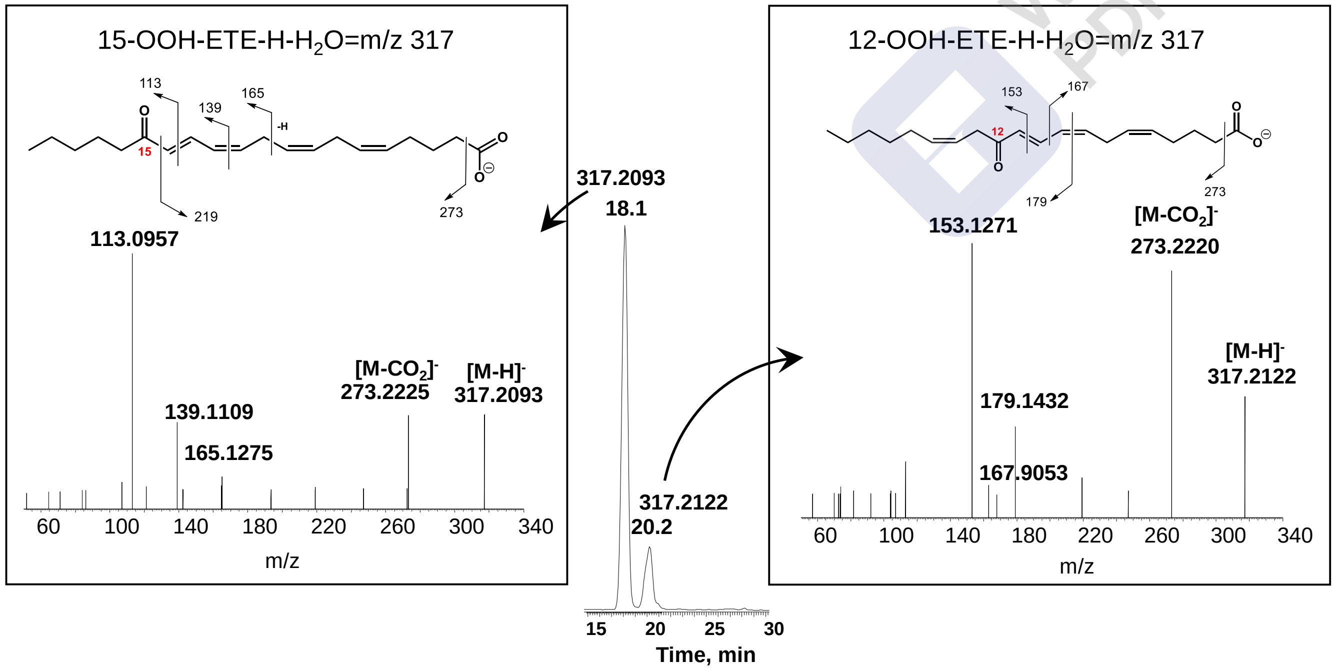

Wondershare PDFelement

Fig 3. Effect of BLX2477 on oxidation of free eicosanotetraenoic acid (ETE) and stearoyl-sn-1-eicosatetraenoyl-sn-1-phosphatidylethanolamine (SA-sn-1-ETE-sn-2-PE) by 15-LOX-1 in the absence and in the presence of PEBP1.

(A) Concentration dependent inhibition of 15LO1 activity by BLX2477 in the presence of ETE (open circles) or in the presence of SA-sn-2-ETE-sn-2-PE (closed circles) (left panel). The data are presented as % of control. 100% of 15LO1 activity in the absence of BLX2477 was used as the control (n=5-7). IC_{50} values of 15LO1 for BLX2477. IC_{50} values were calculated using GraphPad Prism (GraphPad Software, Inc.)

(**B**) 15LO1 oxidation of ETE results in the formation of two oxidized products 15-OOH-ETE and 12-OOH-ETE. Base peak profile of oxygenated ETE formed during 15LO1-driven reaction (middle panel). MS^2 spectrum and structural formulas showing the fragments formed during the analysis of molecular ions with m/z 317.2 corresponding to 15-OOH-ETE without water (left panel) and 12-OOH-ETE without water (right panel).

Experimental conditions: ETE or SA-sn-1-ETE-sn-2-PE were integrated into OA-sn-1-OA-sn-2-PC (100 mM, at ratio of 1:1) liposomes and incubated with 15LO1 (0.4mM) for 2.5 and 5 min at 37°C. Reaction was started by the addition of 3 μM 13 HpODE. At the end of incubation lipids were extracted and analyzed by LC/MS.

透かしを削除する Wondershare PDFelement

Supplemental Figure 4.

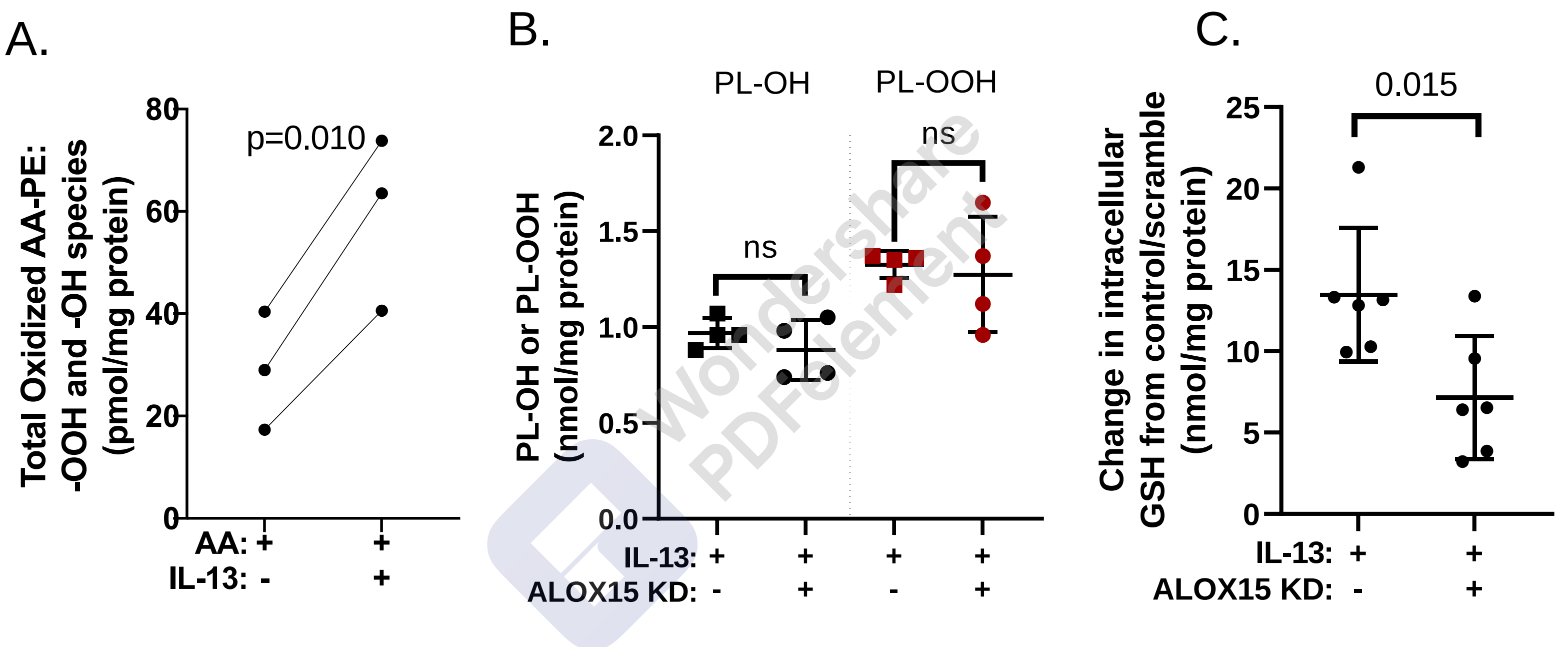

Fig 4. (A) Changes in hydroperoxy- and hydroxy-arachidonic acid (AA) species, including 15-HpETE and 15-HETE, following IL-13 with AA supplementation in HAECs. (**B**) Changes in total hydroperoxy- (PL-OOH) and hydroxy-phospholipids (PL-OH) following IL-13 and ALOX15/15LO1 KD compared to control/scramble in HAECs. (C) Changes in intracellular GSH.

透かしを削除する Wondershare PDFelement

Supplemental Figures.

Fig. 1. GSH and GSSG levels and GSH:GSSG measured by enzymatic method correlated strongly with high-performance liquid chromatography (HPLC) measurements (n=17).

Fig. 2. (A) Representative western blots showing time course of SLC7A11 protein expression in response IL-13 stimulation *in vitro*. (**B**) Representative western bltos showing the inconsistent impact of ALOX15 siRNA knockdown on SLC7A11. (**C**) Densitometric analysis of the fold changes.

Fig 3. Effect of BLX2477 on oxidation of free eicosanotetraenoic acid (ETE) and stearoyl-sn-1-eicosatetraenoyl-sn-1-phosphatidylethanolamine (SA-sn-1-ETE-sn-2-PE) by 15-LOX-1 in the absence and in the presence of PEBP1.

Wondershare PDFelement

(A) Concentration dependent inhibition of 15LO1 activity by BLX2477 in the presence of ETE (open circles) or in the presence of SA-sn-2-ETE-sn-2-PE (closed circles) (left panel). The data are presented as % of control. 100% of 15LO1 activity in the absence of BLX2477 was used as the control (n=5-7). IC_{50} values of 15LO1 for BLX2477. IC_{50} values were calculated using GraphPad Prism (GraphPad Software, Inc.)

(**B**) 15LO1 oxidation of ETE results in the formation of two oxidized products 15-OOH-ETE and 12-OOH-ETE. Base peak profile of oxygenated ETE formed during 15LO1-driven reaction (middle panel). MS^2 spectrum and structural formulas showing the fragments formed

透かしを削除する Wondershare PDFelement

during the analysis of molecular ions with m/z 317.2 corresponding to 15-OOH-ETE without water (left panel) and 12-OOH-ETE without water (right panel).

Experimental conditions: ETE or SA-sn-1-ETE-sn-2-PE were integrated into OA-sn-1-OA-sn-2-PC(100 mM, at ratio of 1:1) liposomes and incubated with 15LO1 (0.4mM) for 2.5 and 5 min at 37°C. Reaction was started by the addition of 3 μM 13 HpODE. At the end of incubation lipids were extracted and analyzed by LC/MS.

Fig 4. (A) Changes in hydroperoxy- and hydroxy-arachidonic acid (AA) species, including 15-HpETE and 15-HETE, following IL-13 with AA supplementation in HAECs. (**B**) Changes in total hydroperoxy- (PL-OOH) and hydroxy-phospholipids (PL-OH) following IL-13 and ALOX15/15LO1 KD compared to control/scramble in HAECs. (**C**) Changes in intracellular GSH.

Wondershare PDFelement

透かしを削除する Wondershare PDFelement

Supplemental Table 1. Characteristics of fresh epithelial cell subset

	HC (n = 11)	M/M (n = 4)	SA (n = 11)	P value
**Age (years)**	44 ± 14	35 ± 18	52 ± 11	0.189
**Female/male**	8/3	2/2	7/4	0.706
**BMI (kg/m^2)**	27 ± 4	28 ± 2	30 ± 7	0.478
**History of exacerbation*, yes/no**	NA	1/3	6/5	0.310
**Inhaled corticosteroids, yes/no**	NA	1/3	11/0	0.001
**ACQ**	NA	0.6 (0.2-1.2)	1.7 (1.3-3.0)	0.023
**AQLQ**	NA	6.3 (5.9-6.7)	5.2 (3.9-5.8)	0.013
**FeNO (ppb)**	11 (9-17)	31 (14-47)	37 (17-45)	0.011
**FEV_1 (% predicted)**	102 ± 13	98 ± 17	65 ± 25	<0.001

Data as means ± SDs

Wondershare PDFelement

透かしを削除する Wondershare PDFelement

p values by ANOVA, Mann-Whitney, Kruskal-Wallis, or χ^2 tests

Exacerbation*, self-reported use of systemic corticosteroid ≥3 days in past 12 months

Wondershare PDFelement

透かしを削除する Wondershare PDFelement

Wondershare PDFelement

透かしを削除する Wondershare PDFelement

Supplemental Table 2. Relationships between extracellular (BAL fluid) and intracellular GSH pathways in asthma and HCs

	BAL fluid		
	GSH (µM)	GSSG (µM)	GSH:GSSG
**Intracellular**			
**GSH (nmol/mg protein)**	**r=0.41**<br>**p=0.037**	r=-0.29<br>p=0.156	**r=0.58**<br>**p=0.002**
**GSSG (nmol/mg protein)**	r=0.26<br>p=0.191	r=-0.09<br>p = 0.645	r=0.33<br>p=0.104
**GSH:GSSG**	r=0.13<br>p=0.517	r= -0.19<br>p= 0.352	r=0.24<br>p=0.234

Wondershare PDFelement

透かしを削除する Wondershare PDFelement

Supplemental Table 3. Non-parametric correlations for BAL eosinophils (absolute, % BAL cells) and GSH, GSSG and GSH:GSSG

Eosinophils

	**Absolute eosinophil numbers**	**% BAL Cells**
BAL Fluid:		
**GSH**	rho=0.02	rho=-0.08
	p=0.877	p=0.417
**GSSG**	rho=-0.01	rho=0.05
	p=0.938	p=0.644
**GSH:GSSG**	rho=-0.05	rho=-0.18
	p=0.610	p=0.078
Intra-epithelial:		
**GSH**	rho=-0.25	rho=-0.33
	p=0.227	p=0.102
**GSSG**	rho=-0.26	rho=-0.29
	p=0.199	p=0.148

Wondershare PDFelement

透かしを削除する Wondershare PDFelement

**GSH:GSSG**	rho=-0.07<br>p=0.730	rho=-0.14<br>p=0.482

Supplemental Table 4. Non-parametric corrleations of inflammatory cells (% BAL cells) versus BAL fluid and intracellular/intra-epithelial GSH, GSSG, and GSH:GSSG.

Other Inflammatory Cells

	Neutrophils (% BAL cells)	Macrophages (% BAL cells)	Lymphocytes (% BAL cells)
BAL Fluid:			
**GSH**	rho=-0.06<br>p=0.586	rho=0.01<br>p=0.989	rho=-0.01<br>p=0.914
**GSSG**	rho=0.16<br>p=0.147	rho=-0.15<br>p=0.156	rho=0.07<br>p=0.542
**GSH:GSSG**	rho=-0.19<br>p=0.089	rho=0.14<br>p=0.206	rho=-0.09<br>p=0.431

Wondershare PDFelement

透かしを削除する Wondershare PDFelement

Intra-epithelial:

**GSH**	rho=-0.07	rho=0.01	rho=0.13
	p=0.725	p=0.985	p=0.527
**GSSG**	rho=0.15	rho=0.17	rho=-0.12
	p=0.481	p=0.403	p=0.557
**GSH:GSSG**	rho=-0.32	rho=0.01	rho=0.07
	p=0.117	p=0.956	p=0.744

Wondershare PDFelement

透かしを削除する Wondershare PDFelement

Supplemental Table 5. Correlation of T2 signature genes and ALOX15 expression.

	**ALOX15 expression**
**CCL26 expression**	**r=0.58**
	**p<0.001**
**MUC5AC expression**	**r=0.41**
	**p<0.001**
**NOS2 expression**	**r=0.67**
	**p<0.001**
**POSTN expression**	**r=0.56**
	**p<0.001**

Bold p-values pass Bonferonni Correction

Wondershare PDFelement
